# Diet of schistosome vectors influences infection outcomes

**DOI:** 10.1002/ecs2.70052

**Published:** 2024-11-18

**Authors:** Joshua Trapp, Wesley Yu, Johannie M. Spaan, Tom Pennance, Fredrick Rawago, George Ogara, Maurice R. Odiere, Michelle L. Steinauer

**Affiliations:** 1 Department of Biomedical Sciences, Western University of Health Sciences, Lebanon, Oregon, USA; 2 Kenya Medical Research Institute (KEMRI), Centre for Global Health Research, Kisumu, Kenya

**Keywords:** *Biomphalaria*, gastropod, nutrition, schistosomiasis, snail

## Abstract

Resource availability can alter infection outcomes through its impact on host immunity and on parasite reproduction. On one hand, enhanced nutrition could favor immunity, limiting the parasite, and on the other hand, it could favor establishment and reproduction of the parasite. Our study aimed to determine the effect of diet on (1) host susceptibility to infection and (2) parasite production in a snail-trematode system. We fed *Biomphalaria sudanica*, a snail vector of *Schistosoma mansoni*, either a strict lettuce (low nutrient) or pellet (high nutrient) diet for two generations before exposing them to *S. mansoni*. We used two parasite strains, one that is incompatible with the snails and one that is compatible with the snails. We found that when exposed to incompatible parasites, diet did not affect snail susceptibility significantly as few snails were infected overall. However, when challenged with the compatible parasites, snails fed the high-nutrient diet were more susceptible to infection than their low-nutrient-fed counterparts. The high-nutrient-fed snails also produced more cercariae than low-nutrient-fed snails, but this advantage was lost after the initial assessment at 8 weeks. Snails that obtained infections were either kept on their initial diet or switched to the other diet. This experiment showed that snails switched from a low-to-high-nutrient diet produced more cercariae than those remaining on the low-nutrient diet and similar numbers to those remaining on the high-nutrient diet. Unexpectedly, the reciprocal diet switch (high to low nutrient) initially resulted in more cercariae relative to controls, but the pattern reversed after initial assessment. This study showed that available resources can impact the susceptibility of the vector host and the reproductive capacity of the parasites, with higher nutrients favoring parasite establishment and reproduction, highlighting the plasticity of susceptibility phenotypes, which also have a strong genetic basis. These data can aid predictions of how future environmental changes and resource availability may impact parasite transmission.

## INTRODUCTION

Resource availability can be a strong driver of infectious disease dynamics through its effects on host–pathogen interactions (Becker & Hall, 2014; Civitello et al., 2018; Cotter & Al Shareefi, 2022). Changes in diet caloric density (quantity) and composition (quality) can have both positive and negative effects on pathogen transmission (Cressler et al., 2014). For instance, increased nutrients could fuel robust immune defenses that are energetically costly, thereby increasing host resistance to pathogen infection (Almire et al., 2021; Seppälä et al., 2022; Sheldon & Verhulst, 1996). Alternatively, increased nutrients could translate to a more successful pathogen that capitalizes on increased energy to fuel reproduction (Becker & Hall, 2014; Rondelaud et al., 2004; Vale et al., 2013). Thus, the outcome of these opposing forces in response to nutrient availability and composition may be difficult to predict. Indeed, infection and immune defense outcomes vary among host and pathogen taxa depending on diet (Cotter & Al Shareefi, 2022), highlighting the need to investigate the effects of diet-related impacts on host-pathogen systems.

Trematodes are a diverse group of flatworm parasites that infect humans, domestic animals, and wildlife. Trematodes have an obligatory mollusk intermediate host (typically an aquatic snail) in which they form chronic infections. The resource-intensive nature of these gastropod infections is evidenced by the abundant parasite biomass generated during this intra-molluscan reproductive stage, which often comprises a significant portion of the total ecosystem biomass (e.g., Kuris et al., 2008; Preston et al., 2013, 2021) making trematodes a significant contributor to ecosystem energetics (Poulin et al., 2013; Vannatta & Minchella, 2021). Because of the resource-intensive nature of parasite reproduction, and the robust immune responses of snails (Pila et al., 2017), resource availability has the potential to impact infection dynamics, influencing human, animal, and ecosystem health (Civitello et al., 2018, 2022; Malishev & Civitello, 2019; Salo et al., 2019; Seppälä et al., 2022; Seppälä & Jokela, 2010).

Of the trematodes, schistosomes have the largest impact on human health, causing a widespread, neglected tropical disease, schistosomiasis, leading to the global loss of over 1.8 million disability adjusted life years (Hay et al., 2017) with 251 million people requiring preventative chemotherapy in 2021 (Kokaliaris et al., 2022). The disease is caused by infection with blood flukes of the genus *Schistosoma* that are transmitted to humans by freshwater snail vectors. Schistosomes chronically infect snails, and following an intramolluscan developmental period, continued asexual reproduction results in the production of hundreds-to-thousands of infectious, free-swimming cercariae that are released daily into freshwater environments throughout the life of the snail (Chu & Dawood, 1970). Cercariae infect humans or other mammals by penetrating the skin and eventually establishing in the vasculature (McManus et al., 2018). Generally, food quantity and quality impact trematode reproduction within snails, with more resources equating to bigger snails and more parasites at the individual level (Belfaiza et al., 2004; Coles, 1973; Desautels et al., 2022). However, snails have an innate immune system that may also be dependent on available resources. Indeed, studies have shown reduced immune function of freshwater snails under nutrient stress (Salo et al., 2019; Seppälä et al., 2022; Seppälä & Jokela, 2010).

To determine the outcome of these apparently opposing factors, we compared the effects of two distinct diets on the infection outcome of snails after exposure to parasites. In the context of our rapidly changing planet, the results of this study further our understanding of how resource availability impacts the population dynamics of trematode parasites, which can facilitate predictions of how ecological changes may influence food webs and how they may also influence disease risk, particularly in the context of snail-based control measures (Civitello et al., 2022).

## METHODS AND MATERIALS

### Parasites and snails

This study used *Biomphalaria sudanica* snails raised in our laboratory and are designated as “KEMRIwu.” This line originated from the Kisumu region of Lake Victoria, Kenya and has been maintained in captivity since 2010. For food, snails were raised on either a strict green leaf lettuce (referred to from here primarily as a “low-nutrient diet”) or Aquatic Blended Foods (ABF) Aquatic Fresh Water Snail Mix, ABF2 formulation (referred to from here primarily as a “high-nutrient diet”) (Appendix S1: Table S1), for two generations and were supplemented with equal amounts of calcium carbonate (essential for healthy shell growth). Food was added to tanks twice per week and amounts adjusted to accommodate the growth of snails over time. Both diets were fed ad libitum so that it was always available; however, availability of pellets was difficult to definitively confirm between feedings because they break down more rapidly than lettuce. Snails were maintained in aquaria, which consisted of 10 L plastic shoe boxes filled with 8 L of aerated and filtered artificial spring water, on shelving in rooms kept at 24–25°C with a light–dark cycle of 12 h.

Snail populations were challenged to two different lines of *Schistosoma mansoni*: Naval Medical Research Institute (NMRI) and UNMKenya. The KEMRIwu snails are compatible with the UNMKenya parasites and incompatible with NMRI parasites (Spaan et al., 2023). NMRI is maintained by the Schistosomiasis Resource Center (Lewis et al., 2008) and was originally obtained from Puerto Rico 50 years ago. NMRI line parasites were obtained from the Schistosomiasis Resource Center via overnight shipment of infected mouse livers containing eggs. UNMKenya was originally collected from Mwea, Kenya (central), in 2013 and maintained in *Biomphalaria pfeifferi* at the University of New Mexico. To introduce more genetic variation to the UNMKenya line, fresh material was added in 2017, representing parasites collected from Asao, Kenya, and maintained in *B. sudanica* and hamster definitive hosts in our laboratory at Western University of Health Sciences. A snail line selected for susceptibility, *B. sudanica* 110S1, was included as a control for NMRI parasite viability during the parasite challenge experiment since the KEMRIwu snail line is resistant to this parasite line (Spaan et al., 2023). 110S1 snails were not assessed after 8 weeks post-exposure.

Project approval received under the relevant bodies, including KEMRI Scientific Review Unit (permit KEMRI/RES/7/3/1), Kenya’s National Commission for Science, Technology, and Innovation (permit NACOSTI/P/22/148/39 and NACOSTI/P/15/9609/4270), Kenya Wildlife Services (permit WRTI-0136-02-22 and permit 0004754), and National Environment, Management Authority (permit NEMA/AGR/159/2022 and permit NEMA/AGR/46/2014) (registration number 0178). The UNMKenya parasite strain was maintained in hamsters at the Western University of Health Sciences under approval of the IACUC (ACUP number R20IACUC039) and IBC (number 23IBC003).

### Snail exposures and assessment of infection

Snails from each diet group with a shell size of 3–4.9 mm (~3–5 months old) were selected from breeding tanks and placed into holding tanks prior to exposure. On the day of exposure, *S. mansoni* eggs were freed from the liver tissue by massaging it through a tissue sieve and rinsing them into a 500-mL Erlenmeyer flask with physiological saline (0.85%). The eggs were rinsed once with saline using the decanting method. Artificial spring water was then added, and the flask exposed to light for 15 min to induce the hatching of miracidia. The bottom of the flask was covered to encourage the photophilic miracidia to swim to the top of the flask allowing for an increased concentration and thus ease of sampling. Five miracidia were pipetted into each well in a 24-well plate and visually confirmed using a stereomicroscope. Each well received 2 mL of artificial spring water, a single snail, and left overnight. The day after exposure, snails were arbitrarily placed into aquaria grouped by diet with a maximum density of 48 snails (this resulted in two replicate tanks per diet group for snails exposed to the incompatible parasite. Snails exposed to compatible parasites had 4 and 2 replicate tanks for lettuce and pellet diets, respectively). A larger sample size was initiated for the lettuce fed snails because the original intention was to perform the diet switch follow-up experiment with only the lettuce fed snails. All exposed snails were maintained as described above with the exception of covered shelving to create a dim environment that reduces early release of cercariae. After 4 weeks, these snails were rehoused into new aquaria with 24 individuals per tank per diet group. Thus, the snails exposed to incompatible parasite had four replicate tanks for each diet group, and snails exposed to the compatible parasite had six and four replicate tanks for lettuce and pellet diets, respectively. Snails exposed to the compatible parasite were assessed for infection at 8, 11, 14, and 17 weeks post-exposure, whereas snails exposed to the incompatible parasite were assessed for infection at 8 and 14 weeks post-exposure due to low infection rates. To assess infection, snails were isolated in wells of a 24-well plate containing 2 mL of artificial spring water and exposed to light for 2 h after which wells were observed via microscopy for the presence of *S. mansoni* cercariae released from infected snails. The number of cercariae produced per snail was estimated by counting the number of cercariae in two 200-μL aliquots of the water after homogenizing with a pipette. To facilitate counting, cercariae were pipetted on to a gridded plate and killed and stained with iodine. The mean of the counts from the two aliquots was multiplied by 10 to achieve an estimated total count. If 10 or fewer cercariae were released from a snail, then a total count of the cercariae in the entire well was performed instead. Snail diameter was measured at the widest width using digital calipers. Non-shedding snails were returned to fresh aquaria tanks and reassessed for infection status at the remaining assessment intervals.

### Snail diet switch

Snails found to be infected with the compatible parasite at 8 weeks post-exposure were randomized into new diet groups in which they were either kept on their same diet or switched to the alternative diet (i.e., low- to high-nutrient diet, or high- to low-nutrient diet) and housed in new aquaria containing only infected snails (single tank per diet switch group). The number of cercariae shed, and snail size was reassessed at 11, 14, and 17 weeks post-exposure using the procedure stated above. Snails were returned to fresh aquaria tanks after each assessment interval.

### Statistical analysis

Most of the analyses focused only on the compatible KEMRIwu—UNMKenya combination of snail and parasites because we found only three infections with the incompatible KEMRIwu—NMRI combination. Prevalence was calculated as the total number of infected snails found across all assessment intervals (8, 11, 14, and/or 17 weeks) divided by the total number of surviving snails at the first assessment (8 weeks) and multiplied by 100. The statistical program R (R Core Team, 2022), including the MASS package (Venables & Ripley, 2002), was used for analysis, and GraphPad Prism version 10.1.0 for macOS, GraphPad Software, Boston, Massachusetts, USA, www.graphpad.com was used for visualization of data.

#### Effect of diet on infection success, parasite production, and snail size

##### Infection success

Infection success (yes/no) was measured by the presence/absence of cercarial shedding. To determine the effect of diet on infection success of the compatible combination of snail and parasite, a generalized linear model (GLM) with binomial family and logit link function was used due to the binary response variable, no violation of independence assumption, and no tank effect was observed (i.e., there was no significant difference in prevalence across replicate tanks for lettuce fed snails [Kruskal–Wallis rank sum test, χ^2^ = 2.2, df = 5, *p* = 0.8253] or pellet fed snails [Kruskal–Wallis rank sum test, χ^2^ = 2.7, df = 3, *p* = 0.4359]; see Appendix S2: Figure S1). Fisher’s exact test was used to determine the effect of diet on infection success, due to low prevalence of the incompatible host–parasite combination.

##### Parasite reproduction

A GLM with a negative binomial distribution and log link function due to over dispersed, count data were used to determine the effect of diet on parasite reproduction (number of cercariae produced) at 8 weeks post-exposure for the compatible KEMRIwu—UNMKenya (host–parasite) combination only. There was no significant difference in the number of cercariae shed across replicate tanks for lettuce fed snails (Kruskal–Wallis rank sum test, χ^2^ = 5.4, df = 5, *p* = 0.3697) or pellet fed snails (Kruskal–Wallis rank sum test, χ^2^ = 1.9, df = 3, *p* = 0.6008) (Appendix S2: Figure S2). While size is a strong predictor of cercarial production in snails (e.g., Spaan et al., 2023), we did not include size in the model because we were interested in the overall effects of diet on cercarial production regardless of the underlying mechanism (see Appendix S3 with additional analysis including snail size as a covariate in the model).

##### Snail size

To determine the effect of diet and infection on snail size at 8 weeks post-exposure, a multiple linear regression model (LM) was used. The two-way interaction between infection status and diet was only included in the final model if statistically significant (*p* < 0.05). Visual inspection of residual plots revealed no violation of the model assumptions (linearity, homoscedasticity, multicollinearity, and independence). There was no significant difference in snail size across replicate tanks for lettuce fed snails (Kruskal–Wallis rank sum test, χ^2^ = 6.2, df = 5, *p* = 0.2858) or pellet fed snails (Kruskal–Wallis rank sum test, χ^2^ = 3.3, df = 3, *p* = 0.3440; Appendix S2: Figure S3).

#### Diet switch effect on parasite production of infection and snail size

##### Parasite production

A GLM with a negative binomial distribution and log link function due to over dispersed, count data was used to determine the effect of diet switch on the cercarial production at each assessment interval (11, 14, and 17 weeks post-exposure) separately. Here, we ran models separately for each assessment interval, as we could not account for the repeated measures of the same individuals. As above, we did not include snail size at time of shedding as a covariate in the models as we were interested in the overall effect of diet including enhanced snail growth (see Appendix S3 for analysis that included size as a covariate). Additionally, sample sizes, particularly at 14 and 17 weeks were inadequate to include size in the model.

##### Snail size

To determine the effect of diet switch on snail size at each assessment interval (11, 14, and 17 weeks post-exposure) separately, a nonparametric Dunn Kruskal–Wallis multiple comparison test was used with adjusted *p* values using Holm’s method (Holm, 1979), due to small sample sizes and non-normally distributed response variables.

## RESULTS

### Effect of diet on infection success, parasite production, and snail size

Snails were highly resistant to infection by the incompatible NMRI parasites, and prevalence was not significantly different between diet groups (Fisher’s exact test, *p* = 0.2465; Table 1). The *B. sudanica* 110S1 control snails displayed a 44% infection prevalence at 8 weeks post-exposure, which was in line with our previous results (Spaan et al., 2023) and indicated that the low prevalence was due to resistance of the snail rather than unviability of the parasite.

**FIGURE 1:**
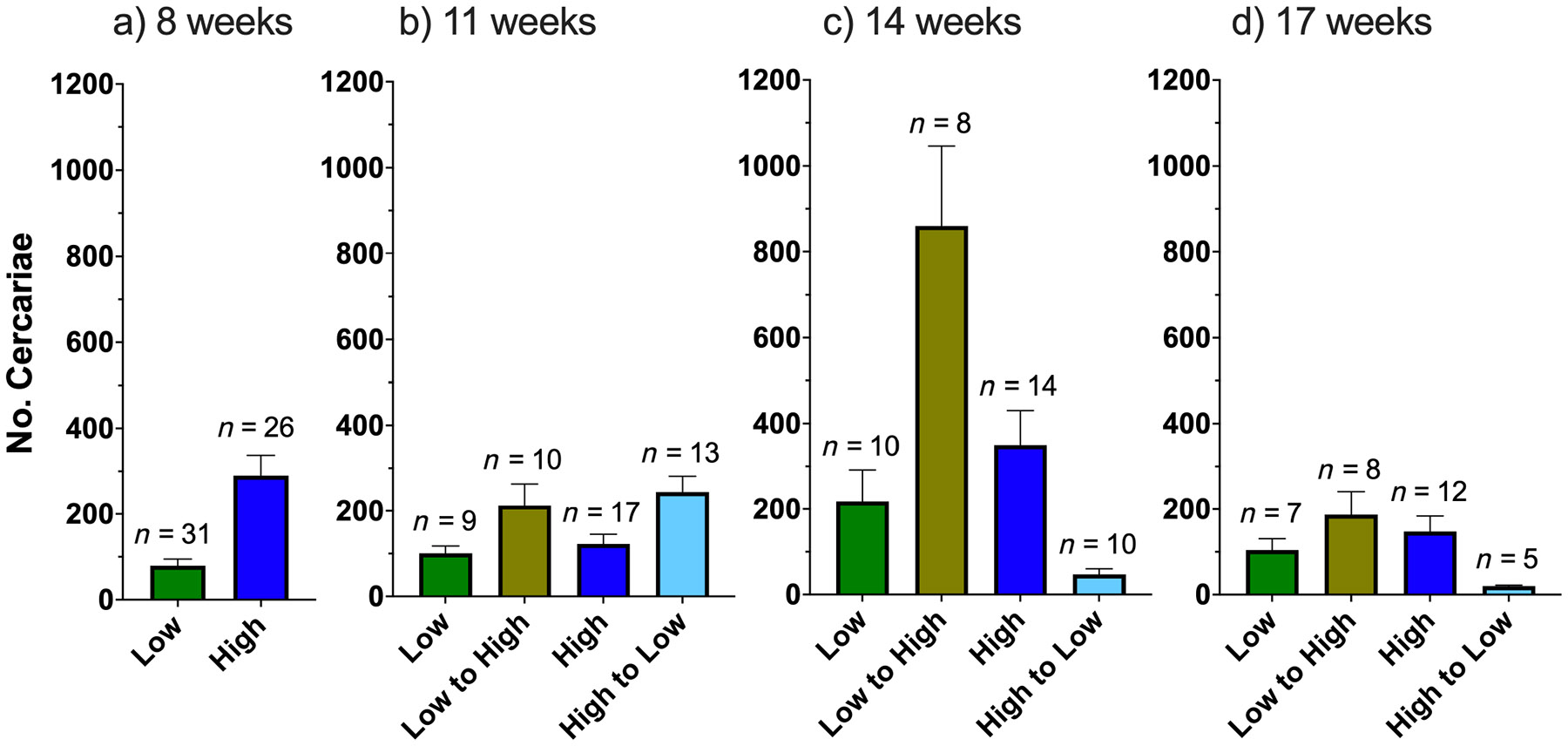
The effect of diet on reproduction of *Schistosoma mansoni* (compatible UNMKenya line) in *Biomphalaria sudanica* (KEMRIwu line) at (a) 8, (b) 11, (c) 14, and (d) 17 weeks post-exposure. After assessment at 8 weeks, the diet of some snails was switched. Error bars represent SE of the mean and numbers represent the sample size per group.

**FIGURE 2:**
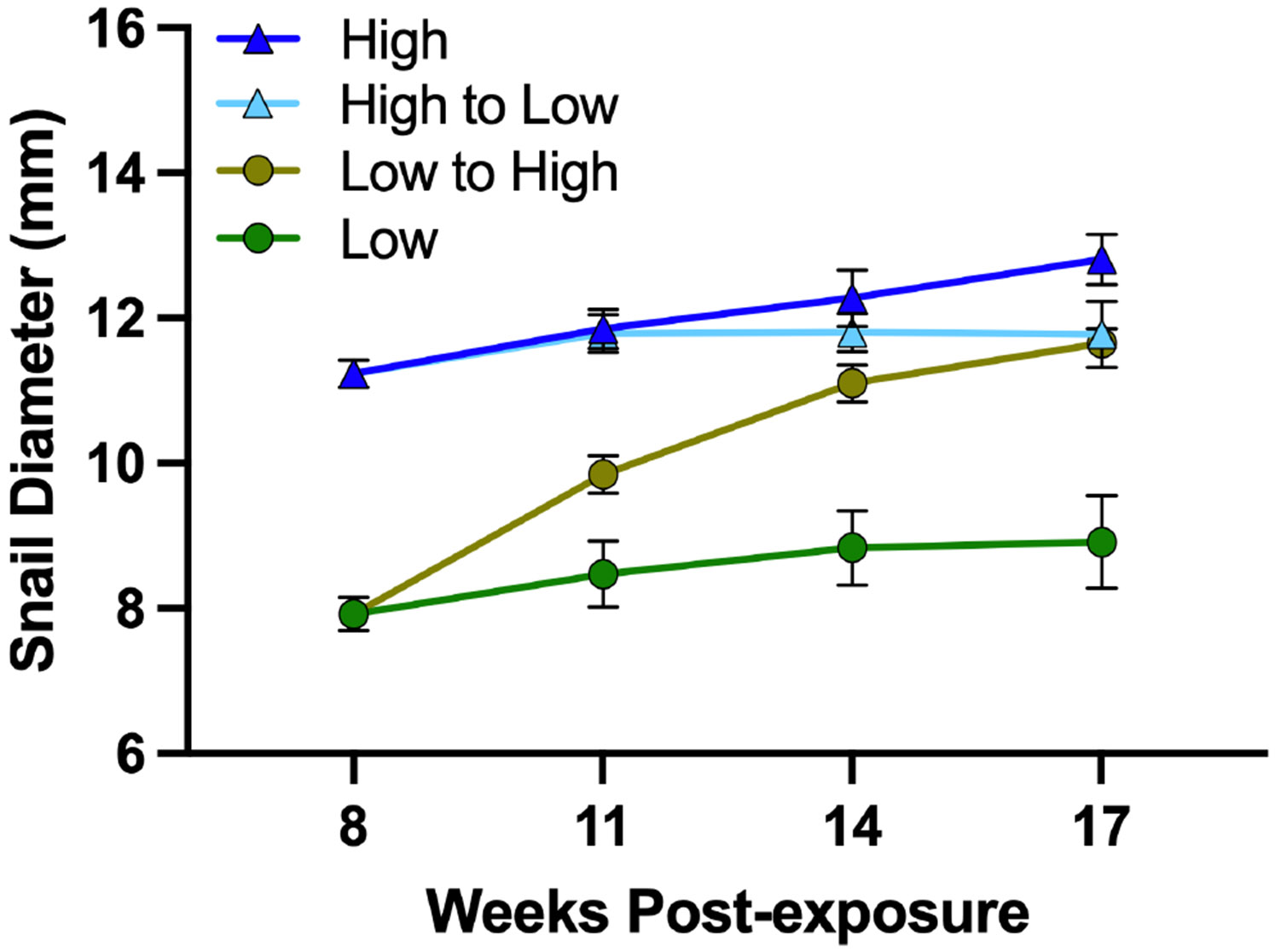
The effect of diet and diet change on the diameter (in millimeters) of *Biomphalaria sudanica* snails infected with *Schistosoma mansoni* (the compatible UNMKenya line) at 8, 11, 14, and 17 weeks post-exposure. Points represent the mean and error bars represent SE of the mean.

When exposed to the compatible parasite, snails fed pellets were found to be more susceptible compared to those fed lettuce, with 101% (CI: 7%–281%) higher odds of infection (GLM, β_0_ = −0.82, β = 0.70, *z* = 2.2, *p* = 0.0304; Appendix S4: Table S1). At 8-weeks post-exposure, pellet-fed snails produced significantly more cercariae (295%) than those fed lettuce (GLM, β_0_ = 4.30, β = 1.37, *z* = 4.9, *p* < 0.0001; Figure 1a; Appendix S4: Table S2) and were significantly larger than lettuce-fed snails, after accounting for infection success (LM, β_0_ = 8.51, β = 2.54, *t* = 14.9, *p* < 0.0001; Figure 2; Appendix S4: Table S3). We performed an exploratory analysis to determine if the differences in parasite production can be attributed to the enhanced growth of the pellet fed snails as size is a strong predictor of parasite production. In this model, we found that size was a significant covariate (GLM, β_0_ = 4.60, β = 0.45, *z* = 2.0, *p* = 0.0463) and diet alone had no significant effects (GLM, β_0_ = 4.60, β = 0.65, *z* = 1.5, *p* = 0.1465; Appendix S3: Table S1). Interestingly, infected snails were larger than snails that resisted infection after accounting for diet (LM, β_0_ = 8.51, β = −0.53, *t* = −3.1, *p* = 0.0025; Appendix S4: Table S3), showing the gigantism phenomenon (e.g., Minchella et al., 1985).

### Diet switch effect on parasite production of infection and snail size

#### Snails switched from a low-nutrient to high-nutrient diet (lettuce to pellets)

Overall, snails switched from the low-to-high-nutrient diet produced significantly more cercariae compared to low-nutrient-fed controls, and statistically similar numbers to high-nutrient-fed controls, supporting the hypothesis that enhanced resources would favor increased parasite reproduction. Specifically, at 11 weeks, snails with the switched diet produced 112% more cercariae than low-nutrient-fed controls (GLM, β_0_ = 4.61, β = 0.75, *z* = 2.2, *p* = 0.0285), and 74% more than high-nutrient-fed controls; however, the difference was not statistically significant (GLM, β_0_ = 4.81, β = 0.55, *z* = 1.8, *p* = 0.0680) (Figure 1b; Appendix S4: Table S4). At 14 weeks post-exposure, the number of cercariae produced by the snails with the switched diet (low to high nutrient) peaked and was higher than any other diet group (Figure 1c). They produced 294% more cercariae than low-nutrient-fed controls (GLM, β_0_ = 5.39, β = 1.37, *z* = 2.8, *p* = 0.0052), and 146% more cercariae than high-nutrient-fed controls (GLM, β_0_ = 5.86, β = 0.90, *z* = 1.9, *p* = 0.0507) (Figure 1c; Appendix S4: Table S4). By 17 weeks, the number of cercariae produced from snails with the switched diet (low to high nutrient) was similar to both controls; however, sample sizes were small due to mortality (switched diet vs. low-nutrient diet: GLM, β_0_ = 4.65, β = 0.59, *z* = 1.4, *p* = 0.1489) (switched diet vs. high-nutrient diet: GLM, β_0_ = 4.99, β = 0.24, *z* = 0.7, *p* = 0.5070) (Figure 1d; Appendix S4: Table S4). Growth of snails with the switched diet increased significantly relative to low-nutrient-fed controls (Dunn’s test, 11 weeks: *p* = 0.2686; 14 weeks: *p* = 0.0574; 17 weeks: *p* = 0.0396) and by 14 weeks, these snails were of similar size to those fed the high-nutrient diet throughout the experiment (Dunn’s test, 11 weeks: *p* = 0.0023; 14 weeks: *p* = 0.0931; 17 weeks: *p* = 0.1476; Figure 2, Table 2).

#### Snails switched from a high-nutrient to low-nutrient diet (pellets to lettuce)

Surprisingly, the number of cercariae produced by snails switched from a high-to-low-nutrient diet spiked after the diet switch but then sharply declined over time (Figure 1). At 11 weeks post-exposure these snails produced 99% more cercariae than the high-nutrient-fed controls (GLM, β_0_ = 4.81, β = 0.69, *z* = 2.5, *p* = 0.0127) and 143% more than low-nutrient-fed controls (GLM, β_0_ = 4.61, β = 0.89, *z* = 2.8, *p* = 0.0056) (Figure 1b; Appendix S4: Table S4). At 14 weeks post-exposure, snails switched from a high-to-low-nutrient diet produced the fewest number of cercariae: 78% fewer cercariae than low-nutrient-fed controls (GLM, β_0_ = 5.39, β = −1.53, *z* = −3.2, *p* = 0.0014) and 87% fewer cercariae than high-nutrient-fed controls (GLM, β_0_ = 5.86, β = −2.00, *z* = −4.5, *p* < 0.0001; Figure 1c; Appendix S4: Table S4). At 17 weeks, survivorship was poor (only five remaining snails in the high-to-low-nutrient diet) and each of the survivors produced 81% fewer cercariae than the low-nutrient-fed controls (GLM, β_0_ = 4.65, β = −1.65, *z* = −3.4, *p* = 0.0006), and 86% fewer than high-nutrient controls (GLM, β_0_ = 4.99, β = −1.99, *z* = −4.5, *p* < 0.0001; Figure 1d; Appendix S4: Table S4). Snails with the switched diet (high to low nutrient) remained of similar size as high-nutrient-fed controls (Dunn’s test, 11 weeks: *p* = 0.4402; 14 weeks: *p* = 0.2786; 17 weeks: *p* = 0.1052) but were significantly larger than low-nutrient-fed controls. At 17 weeks post-exposure, differences in size were no longer statistically significant, likely due to low sample size (Dunn’s test, 11 weeks: *p* = 0.0001; 14 weeks: *p* = 0.0011; 17 weeks: *p* = 0.1269; Figure 2, Table 2).

#### Comparison of control groups: Low nutrient versus high nutrient (lettuce vs. pellet)

The number of cercariae produced by the control groups were not significantly different from each other at each assessment period post-diet switch, indicating that the advantage gained by the pellet diet was lost after 8 weeks (GLM, 11 weeks: β_0_ = 4.81, β = −0.20, *z* = −0.7, *p* = 0.5131; 14 weeks: β_0_ = 5.86, β = −0.47, *z* = −1.1, *p* = 0.2907; 17 weeks: β_0_ = 4.99, β = −0.35, *z* = −0.9, *p* = 0.3560; Figure 1; Appendix S4: Table S4). Snails fed only pellets did retain their size advantage, being significantly larger than their lettuce-fed counterparts at each assessment interval (Dunn’s test, 11 weeks: *p* < 0.0001; 14 weeks: *p* < 0.0001; 17 weeks: *p* = 0.0001; Figure 2, Table 2).

While snail size was not included in the above models due to our main question regarding diet and our lower sample sizes at 14 and 17 weeks, we explored the effect of snail growth on cercarial production by including size in the models. The relevance of snail size at time of shedding to cercarial production was dependent on the time point considered. Snail size at time of shedding was relevant to cercarial production at 8 weeks (as explained above; Appendix S3: Table S1), 14 weeks (GLM, β_0_ = 5.37, β = 0.65, *z* = 2.6, *p* = 0.0088; Appendix S3: Table S2), and 17 weeks (GLM, β_0_ = 4.48, β = 0.66, *z* = 2.9, *p* = 0.0043; Appendix S3: Table S2), but not 11 weeks (GLM, β_0_ = 4.62, β = 0.29, *z* = 1.6, *p* = 0.1078; Appendix S3: Table S2).

## DISCUSSION

### High-nutrient diet increases snail host susceptibility

The results of this study underscore the resource-dependent nature of schistosomiasis, in terms of parasite establishment and reproduction within the snail. In our experiment, snails fed the high-nutrient pellet diet were 101% more likely to be infected than their low-nutrient-fed counterparts after exposure to a line of *S. mansoni* with which they are susceptible. However, when exposed to an incompatible line of *S. mansoni*, there was no difference in prevalence between the diet groups. Thus, while diet can influence susceptibility levels, it does not appear to reverse the resistance phenotype. It should be noted that the pellet-fed snails did acquire numerically more infections than their lettuce-fed counterparts; however, due to the low number of infections, the increase was not statistically significant. To our knowledge, nutrition-dependent susceptibility of *Biomphalaria* snails to schistosomes has not been previously reported.

Our results suggest that during the initial encounter of the snail and parasite, enhanced resources tip the scale toward the parasite infectivity rather than the snail immunity, resulting in a higher parasite establishment rate. This is the opposite pattern to what was expected since, in previous studies of the freshwater snail *Lymnaea stagnalis*, a high-protein diet increased the immune defense (Seppälä et al., 2022), whereas starvation decreased immune defense (Seppälä & Jokela, 2010). These results suggest some plasticity in the host–parasite interaction and that the outcome of the encounter can be influenced by resource availability. However, there are two potential alternative explanations that would not invoke plasticity in the response: (1) the high-nutrient diet supports greater survival of infected snails than the low-nutrient diet and (2) the difference is driven by differential rates of host finding presuming that the two diets yield different attractants or levels of attractants for the parasites. While we cannot rule out differential survival of infected snails, our data do not support this hypothesis. First, survival was nearly identical between the two diet groups (Table 1; Appendix S5: Table S1). Second, if differential survival of snails based on infection was occurring, we would expect to see a significant relationship among survivorship and prevalence in our replicate tanks so that tanks with lower survivorship would have lower prevalence. Our data showed no such relationship (Appendix S5: Table S2, Figure S1). The second alternative explanation is derived from the observation that some candidate attractants of *Biomphalaria glabrata* are peptides associated with feeding (Fogarty et al., 2019; Macinnis et al., 1974), and thus, miracidia could be more attracted to snails on certain diets. However, given the small size of the arena in which exposures occurred in our experiment (24 well plates), it is unclear whether differential host signal production would be relevant for parasite seeking in this study. Furthermore, in a preliminary host seeking trial, with the same line of *B. sudanica* and the same parasites, we found that parasites found starved and fed snails at equal rates (Appendix S6).

### Higher nutrient diet of host increases parasite reproduction

In addition to increased prevalence, the higher nutrient diet also led to increased *S. mansoni* reproduction within *B. sudanica* at 8 weeks post-exposure, an observation consistent with bioenergetic theory (Civitello et al., 2018). Diet quality or quantity has previously been shown to increase trematode parasite production in snails in the *S. mansoni–B. glabrata* system as well as with other species of snails and trematodes, indicating that the diet effect on parasite production may be a more generalizable trait across snails and trematode systems (Belfaiza et al., 2004; Coles, 1973; Desautels et al., 2022; Sandland & Minchella, 2003). As with previous studies (Mutuku et al., 2021; Spaan et al., 2022, 2023; Tavalire et al., 2016), the increased growth of snails due to diet appeared to be the primary mechanism for enhanced parasite reproduction. However, our exploratory analyses suggest that post 8-weeks, snail size is not always a significant factor in cercarial production and the diet changes, also had significant effects (Appendix S3).

Interestingly, the effects of lettuce and pellet diets on parasite reproduction did not continue after the initial assessment at 8 weeks, since snails remaining on these diets no longer differed in the numbers of cercariae produced even though their sizes and nutrient input remained significantly different throughout the remainder of the experiment (up to 17 weeks post-exposure).

### Effects of diet switch

The diet switch experiment, in which snails assigned to one diet group were randomly reassigned to either the alternative diet or the same diet (control), corroborated our result that the high-nutrient diet resulted in an increase in both snail size and parasite reproduction. These results suggest that any deficits that may have been induced by the low-nutrient diet early in development were quickly reversed with diet change. This experiment also showed that switching snails from the high-nutrient to low-nutrient diet eventually led to a reduction in cercarial production compared to controls. However, at the first assessment post-diet-switch (3 weeks), these snails produced higher numbers of cercariae than the control groups. This result was unexpected, and we can only hypothesize regarding mechanisms underlying the increase in parasite reproduction despite reduced nutrient resources. It is possible that the peak in parasite reproduction was triggered by a stress response of the snail, which cued a rapid increase in parasite reproduction to maximize fitness due to a potential increased probability of host death (e.g., Hechinger, 2010). Alternatively, the increased spike in parasite reproduction despite limited nutrients may be the result of diet diversity and its effects on the composition of the gut microbiome which enhance digestion, particularly plant materials such as cellulose (Cardoso et al., 2012; Harris et al., 2019; Hu et al., 2018). For instance, previous studies comparing gut microbiomes of the snails fed on different diets showed distinctly different microbiomes with differential abilities to digest plant materials (Du et al., 2022; Hu et al., 2021). While more work is needed to understand this unexpected result, this observation suggests that the outcome of differential diet inputs may not follow a simple bioenergetic model.

Although not quantified as a part of this study, infected snail reproduction differed dramatically between the nutrient groups, which was evident after they were moved to new aquaria at 8 weeks for the diet switch experiment. Particularly striking was the large numbers of egg masses and juvenile snails produced by infected snails fed the high-nutrient diet compared to the limited numbers present in infected snails fed the lettuce diet (control groups). Schistosomes castrate their snail hosts, and thus, reproduction is quite limited in laboratory experiments (Etges & Gresso, 1965; Faro et al., 2013). However, it appears that with the added resources from the pellet diet, snails were able to maintain some level of reproduction.

## CONCLUSIONS

Two key novel findings from this study are the demonstration that snail susceptibility is somewhat plastic and that is altered by diet and that snail diet change leads to a surge in parasite reproduction, even if switching from a high- to low-nutrient diet. This study also corroborated previous work showing that nutrient rich diets of snails can lead to an increase in trematode parasite production, at least initially. Together, these findings support the growing evidence of the important links between ecology and infectious disease risk (e.g., Civitello et al., 2022), which is critical in an era of unprecedented levels of ecologic change. Environmental stressors such as climate change, pollution, and eutrophication are predicted to affect both quantity and quality of resource nutrients, biodiversity, and food webs (Birk et al., 2020; Rosenblatt & Schmitz, 2016; Wang et al., 2021) and thus could have a large impact on snail food sources. Furthermore, inadvertent provisioning of snails through aquaculture may impact trematode local transmission dynamics and human infection risk.

## Supplementary Material

Appendix s1

Appendix s2

Appendix S3

Appendix S6

Appendix S4

Appendix S5

## Figures and Tables

**TABLE 1:** Summary of the experimental details including *Schistosoma mansoni* lines (Naval Medical Research Institute [NMRI] or UNMKenya), diet for each group of *Biomphalaria sudanica* snail (green leaf lettuce [low-nutrient level] or commercial pellet [high-nutrient level]), total number of *B. sudanica* snails exposed in each category, total number of snails surviving at 8 weeks post-exposure, and the number of new infections found at each assessment interval (8, 11, 14, and 17 weeks).

Schistosome line	Incompatible NMRI		Compatible UNMKenya	
	Low	High	Low	High
Snails exposed	96	96	141	92
Snails survived to 8 weeks	67 (69%)	74 (73%)	101 (72%)	66 (72%)
No. *S. mansoni* infected snails found at				
8 weeks	0	3	26	31
11 weeks	…	…	3	0
14 weeks	0	0	4	0
17 weeks	…	…	0	0
Total snails infected (prevalence)	0	3 (4%)	33 (33%)	31 (47%)

**TABLE 2:** The effect of diet, and diet change on the diameter of *Biomphalaria sudanica* snails infected with *Schistosoma mansoni* at three assessment intervals (11, 14, and 17 weeks post-exposure to *S. mansoni*).

Comparisons (dietary nutrition):	11 weeks post-exposure			14 weeks post-exposure			17 weeks post-exposure		
	$\bar{X}$	*z*	Adjusted *p*	$\bar{X}$	*z*	Adjusted *p*	$\bar{X}$	*z*	Adjusted *p*
Low control versus high control	−3.38	−4.5	**<0.0001**	−3.44	−4.3	**<0.0001**	−3.89	−4.2	**0.0001**
Low to high versus low control	1.37	1.1	0.2686	2.27	2.2	0.0574	2.74	2.4	**0.0396**
Low to high versus high control	−2.00	−3.3	**0.0023**	−1.18	−1.9	0.0931	−1.15	−1.7	0.1476
High to low versus high control	−0.06	−0.2	0.4402	−0.48	−0.6	0.2786	−1.04	−1.6	0.1052
High to low versus low control	3.32	4.1	**0.0001**	2.97	3.5	**0.0011**	2.86	1.9	0.1269
Low to high versus high to low	−1.94	−2.9	**0.0049**	−0.70	−1.2	0.2225	−0.12	0.2	0.4091

*Note*: Data were analyzed using Dunn’s test (Kruskal–Wallis multiple comparisons) and *p* values are adjusted according to Holm’s method (Holm, 1979); *p* values in boldface represent those <0.05. Low-dietary nutrition represents snails fed green leaf lettuce, whereas high dietary nutrition represents snails fed commercially available pellets (or Aquatic Blended Foods Aquatic Fresh Water Snail Mix).

## Data Availability

Data (Trapp et al., 2024) are available from Figshare: https://doi.org/10.6084/m9.figshare.27146502.v1.
