# Supplementary material for: Diet of schistosome vectors influences infection outcomes
Source: Ecosphere. Author manuscript; Available in PMC 2025 Sep 17. (PMC12439756; doi:10.1002/ecs2.70052)
Supplement: Appendix s1 [file NIHMS2070845-supplement-Appendix_s1.pdf]

Joshua Trapp, Wesley Yu, Johannie M. Spaan, Tom Pennance, Fredrick Rawago, George Ogara, Maurice R. Odiere, Michelle Steinauer. Diet of schistosome vectors influences infection outcomes. Ecosphere.

Appendix S6: Exploring the hypothesis that parasites differentially find and penetrate snails that are recently fed compared to starved snails

Purpose: One potential explanation for the observed difference in infection rates between snails fed pellets and snails fed lettuce is that parasites cue in on snail signals that play a role in feeding. Previous work by Miura et al (2022) showed that *Schistosoma mansoni* miracidia were attracted to snail conditioned water (SCW) from *Biomphalaria glabrata* that were recently fed, but not to SCW from *B. glabrata* that were starved. We performed a similar experiment with *B. sudanica* (KEMRIwu) exposed to *S. mansoni* (compatible UNMKenya line). The experimental design compared numbers of penetrating miracidia in snails that were starved for 7 days compared to those that were fed *ad libitum* with lettuce supplemented by pellets.

Assay Design: To infer the rate of penetration, we individually exposed *B. sudanica* (KEMRIwu) snails (4-4.9 mm) to 8 miracidia in the wells of a 24 well tissue culture plate, took the snails out after 30 min, and counted the remaining miracidia. Control wells to which miracidia, but no snail, were added to assess our counting success rate.

The experiment was set up in replicates of 6 wells for each treatment group and the order was randomized within replicates. Miracidia were pipetted into the wells using a micropipette and stereomicroscope, then recounted to ensure exactly 8 were pipetted into each well. Each well also received 2 mL of artificial spring water prior to introduction of a snail. After the snails were removed, the wells were exhaustively searched for active, misshapen, and dead miracidia. The counter was blind to the treatment groups. A one-way ANOVA with multiple comparisons with Tukey's corrections was used to determine differences among starved, fed, and control wells.

Findings:

Results showed that treatment was significant and that while both starved and fed snails were different from controls, they were not different to each other (Table S1, S2, Figure S1). Also, all snails were found to be penetrated by at least 2 miracidia in 30 minutes post exposure.

Table S1. Ordinary one-way ANOVA results comparing the numbers of miracidia that penetrated among treatment groups (control, fed, or starved).

	SS	DF	MS	$F_{(2, 45)}$	p
Treatment	45.03	2	22.52	20.92	<0.0001
Residual	48.44	45	1.077		
Total	93.48	47			

Table S2. Ordinary one-way ANOVA multiple comparisons to determine differences among treatment groups. Tukey's multiple comparisons test was employed. Note that

while there was no difference between starved and fed snails, both groups differed significantly from controls.

Comparisons:	Mean Diff.	95% CI of diff.	Adjusted *p*-value
Control vs. Starved	-2.944	-4.130 to -1.759	<0.0001
Control vs. Fed	-2.917	-4.064 to -1.769	<0.0001
Starved vs. Fed	0.02778	-0.7563 to 0.8119	0.9959

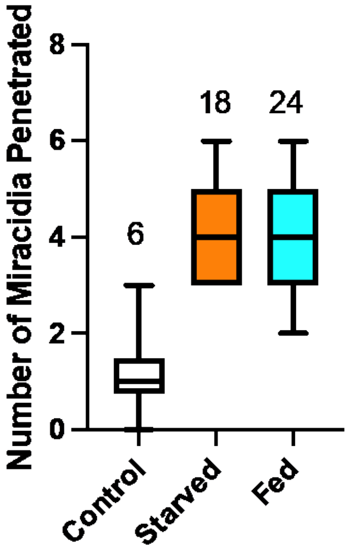

Figure S1. Number of miracidia of *Schistosoma mansoni* (compatible UNMKenya line) out of 8 total that penetrated *Biomphalaria sudanica* (KEMRIwu) snails in 30 minutes. Snails were either starved for 7 days or fed ad libitum. Control wells contained no snails.

Conclusion: Difference in snail feeding signals to not affect the rate at which miracidia penetrated snails in the context of our infection arenas. All snails, regardless of feeding status were found to have been penetrated by at least 2 miracidia. Therefore, it is unlikely that differential penetration rates between lettuce and pellet fed snails are driving the overall differences in infection prevalence rates.

Literature Cited

Miura, M. Y. Mitsui, Y. Aoki, K. Kato. 2022. A new accumulation assay of *Schistosoma mansoni* miracidia using square capillary glass tubes. Experimental Parasitology 239: 108313. ISSN 0014-4894. https://doi.org/10.1016/j.exppara.2022.108313.
