# Supplementary material for: Diet of schistosome vectors influences infection outcomes
Source: Ecosphere. Author manuscript; Available in PMC 2025 Sep 17. (PMC12439756; doi:10.1002/ecs2.70052)
Supplement: Appendix s2 [file NIHMS2070845-supplement-Appendix_s2.pdf]

Joshua Trapp, Wesley Yu, Johannie M. Spaan, Tom Pennance, Fredrick Rawago, George Ogara, Maurice R. Odiere, Michelle Steinauer. Diet of schistosome vectors influences infection outcomes. Ecosphere.

## Appendix S5: Exploring the relationship between survival and prevalence

**Purpose:** One potential explanation for the observed difference in infection rates between snails fed pellets and snails fed lettuce is that survivorship of infected snails was lower prior to assessment at 8 weeks compared to pellet-fed snails. If this were the case, we would expect the following:

1. Overall survival would be lower in lettuce fed snails compared to pellet fed snails.
2. For lettuce fed snails, there should be an association between survivorship rates and infection rates in replicate tanks.

**Findings:**

1. Overall survivorship was the same between lettuce fed and pellet fed snails at 8 weeks (Table S1).

**Table S1.** Number of *Biomphalaria sudanica* snails exposed to *Schistosoma mansoni* (compatible UNMKenya line) and number surviving to 8 weeks. Snails were either fed a lettuce (low nutrient) or pellet (high nutrient) diet.

| | # Surviving | # Exposed | Percent |
|---|---|---|---|
| Low | 101 | 141 | 72% |
| High | 66 | 92 | 72% |

2. Snails were housed in replicate tanks. If infected lettuce fed snails were more likely to die before 8 weeks than uninfected lettuce fed snails, we would expect a positive correlation. We performed Spearman's rank Correlation for the lettuce and pellet tanks and found no significant correlation despite a large variance in survivorship among tanks (Figure S1, Table S2).

**Table S2.** Results of Spearman's Rank Correlation of survivorship and prevalence in replicate tanks for lettuce and pellet fed *Biomphalaria sudanica* snails exposed to *Schistosoma mansoni* (compatible UNMKenya line).

| | Spearman rho | *p*-value |
|---|---|---|
| Low | 0.08 | 0.46 |
| High | -0.4 | 0.38 |

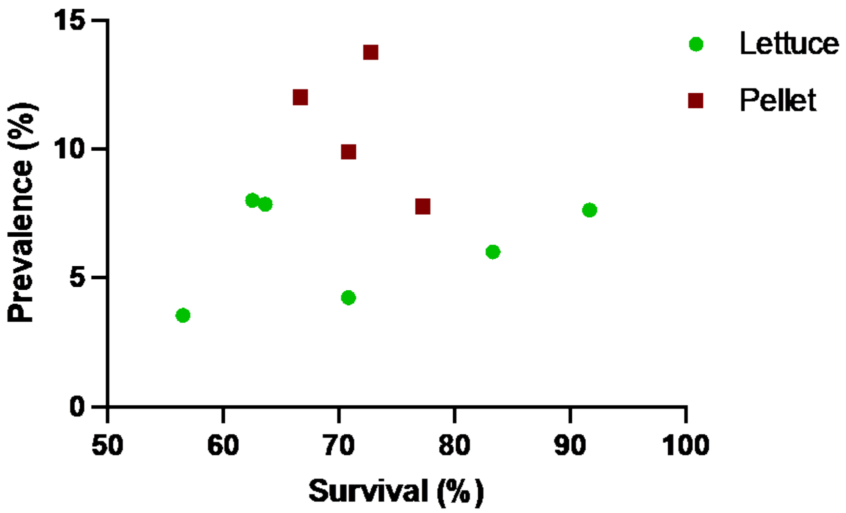


**Figure S1.** Relationship between *Biomphalaria* sudanica snail survival and infection prevalence compared among replicate tanks fed either a lettuce or pellet diet.

**Conclusion:** While we cannot rule out the possibility that a survival differential resulted in the observed difference in prevalence rates between infected and uninfected snails, the evidence does not support this hypothesis.
