# Supplementary material for: Diet of schistosome vectors influences infection outcomes
Source: Ecosphere. Author manuscript; Available in PMC 2025 Sep 17. (PMC12439756; doi:10.1002/ecs2.70052)
Supplement: Appendix S3 [file NIHMS2070845-supplement-Appendix_S3.pdf]

Joshua Trapp, Wesley Yu, Johannie M. Spaan, Tom Pennance, Fredrick Rawago, George Ogara, Maurice R. Odiere, Michelle Steinauer. Diet of schistosome vectors influences infection outcomes. Ecosphere.

**Appendix S4**

**Purpose:** Details of statistical results.

**Table S1:** Summary of the generalized linear model output with a binomial family, logit link function to determine the effect of *Biomphalaria sudanica* KEMRIwu diet (snails fed lettuce vs. pellet) on infection status (shedding positive or negative) when exposed to *Schistosoma mansoni* (compatible UNMKenya line). Odds ratios represents the back transformed estimates ($\beta$). Reference level for diet is lettuce.

| | Estimate ($\boldsymbol{\beta}$) ± SE | Odds ratio (CI's) | *z*-value | *p*-value |
|---|---|---|---|---|
| Intercept ($\boldsymbol{\beta}_0$) | -0.82 ± 0.21 | 0.44 (0.29 – 0.66) | -3.9 | <0.0001 |
| Diet: High | 0.70 ± 0.32 | 2.01 (1.07 – 3.81) | 2.2 | **0.0304** |

**Table S2:** Summary of the generalized linear model output with a negative binomial family, log link function to determine the effect of *Biomphalaria sudanica* KEMRIwu diet (snails fed lettuce vs. pellet) on parasite production (number of cercariae produced) when exposed to *Schistosoma mansoni* (compatible UNMKenya line). Odds ratios represents the back transformed estimates ($\beta$). Reference level for diet is lettuce.

| | Estimate ($\boldsymbol{\beta}$) ± SE | Odds ratio (CI's) | *z*-value | *p*-value |
|---|---|---|---|---|
| Intercept ($\boldsymbol{\beta}_0$) | 4.30 ± 0.20 | 73.33 (50.98 – 110.78) | 21.8 | <0.0001 |
| Diet: High | 1.37 ± 0.28 | 3.95 (2.27 – 6.92) | 4.9 | **<0.0001** |

**Table S3:** Summary of the linear model output to determine the effect of *Biomphalaria sudanica* KEMRIwu diet (snails fed lettuce vs. pellet) and infection status (yes/no) when exposed to *Schistosoma mansoni* (compatible UNMKenya line) on snail size (mm). Reference level for diet is lettuce and infection status are yes.

| | Estimate ($\boldsymbol{\beta}$) ± SE | *t*-value | *P*-value |
|---|---|---|---|
| Intercept ($\boldsymbol{\beta}_0$) | 8.51 ± 0.16 | 53.5 | <0.0001 |
| Infection status: No | -0.53 ± 0.17 | -3.1 | **0.0025** |
| Diet: High | 2.54 ± 0.17 | 14.9 | **<0.0001** |

**Table S4:** Summary of the generalized linear model outputs with a negative binomial family, log link function to determine the effect of compatible *Schistosoma mansoni* (UNMKenya line) infected *Biomphalaria sudanica* snails whose diet (lettuce or pellet) got either switched (lettuce to pellet or pellet to lettuce) or remained on the same diet (lettuce control or pellet control) on parasite production (number of cercariae produced) at three different assessment intervals (11-, 14-, and 17-weeks post exposure). Low dietary nutrition represents snails fed green leaf lettuce, whereas high dietary nutrition represents snails fed commercially available pellets (or Aquatic Blended Foods Aquatic Fresh Water Snail Mix). Abbreviations: $\beta_0$ = Intercept, Est.= Estimate, SE = standard error, OR = odds ratio, CI's = 95 % confidence intervals.

| | **11 weeks post exposure** | | | | **14 weeks post exposure** | | | | **17 weeks post exposure** | | | |
|---|---|---|---|---|---|---|---|---|---|---|---|---|
| **Comparisons (dietary nutrition):** | $\beta_0$ | **Est. ($\beta$) ± SE** | **OR (CI's)** | ***p*-value (*z*-value)** | $\beta_0$ | **Est. ($\beta$) ± SE** | **OR (CI's)** | ***p*-value (*z*-value)** | $\beta_0$ | **Est. ($\beta$) ± SE** | **OR (CI's)** | ***p*-value (*z*-value)** |
| low control vs. high control | 4.81 | -0.20 ± 0.30 | 0.82 (0.46 – 1.52) | 0.5131 (-0.7) | 5.86 | -0.47 ± 0.45 | 0.62 (0.26 – 1.54) | 0.2907 (-1.06) | 4.99 | -0.35 ± 0.38 | 0.71 (0.34 – 1.52) | 0.3560 (-0.9) |
| low to high vs. low control | 4.61 | 0.75 ± 0.34 | 2.12 (1.08 – 4.17) | **0.0285** (2.2) | 5.39 | 1.37 ± 0.49 | 3.94 (1.49 – 10.59) | **0.0052** (2.8) | 4.65 | 0.59 ± 0.41 | 1.80 (0.80 – 3.99) | 0.1489 (1.4) |
| low to high vs. high control | 4.81 | 0.55 ± 0.30 | 1.74 (0.97 – 3.21) | 0.0680 (1.8) | 5.86 | 0.90 ± 0.46 | 2.46 (1.01 – 6.34) | **0.0507** (1.9) | 4.99 | 0.24 ± 0.36 | 1.27 (0.63 – 2.62) | 0.5070 (0.7) |
| high to low vs. high control | 4.81 | 0.69 ± 0.28 | 1.99 (1.16 – 3.47) | **0.0127** (2.5) | 5.86 | -2.00 ± 0.45 | 0.13 (0.06 – 0.33) | **<0.0001** (-4.5) | 4.99 | -1.99 ± 0.44 | 0.14 (0.06 – 0.34) | **<0.0001** (-4.5) |
| high to low vs. low control | 4.61 | 0.89 ± 0.32 | 2.43 (1.28 – 4.54) | **0.0056** (2.8) | 5.39 | -1.53 ± 0.48 | 0.22 (0.08 – 0.56) | **0.0014** (-3.2) | 4.65 | -1.65 ± 0.48 | 0.19 (0.08 – 0.52) | **0.0006** (-3.4) |
| low to high vs. high to low | 5.50 | -0.14 ± 0.32 | 0.87 (0.47 – 1.65) | 0.6647 (-0.4) | 3.85 | 2.90 ± 0.49 | 18.25 (6.89 – 49.27) | **<0.0001** (5.9) | 3.00 | 2.24 ± 0.47 | 9.36 (3.56 – 22.97) | **<0.0001** (4.8) |
