# Supplementary material for: Diet of schistosome vectors influences infection outcomes
Source: Ecosphere. Author manuscript; Available in PMC 2025 Sep 17. (PMC12439756; doi:10.1002/ecs2.70052)
Supplement: Appendix S6 [file NIHMS2070845-supplement-Appendix_S6.pdf]

Joshua Trapp, Wesley Yu, Johannie M. Spaan, Tom Pennance, Fredrick Rawago, George Ogara, Maurice R. Odiere, Michelle Steinauer. Diet of schistosome vectors influences infection outcomes. Ecosphere.

Appendix S1

Table S1. Summary of the macronutrients for both Lettuce (ars.usda.gov) and the ABF Super Freshwater Snail Mix #2 (www.aquaticblendedfoods.com).

Nutritional Value of Snail Diet per 1 gram					
		ABF Super Freshwater Snail Mix # 2			
Nutritional Value	Lettuce	Premium Krill Bits	Super Veggie Sticks with Calcium, Spirulina and Garlic	ABF Premium Mini Veggie Sticks with Calcium	ABF Gourmet Mini shrimp and snail sticks with spirulina, Krill and Calcium
Protein (g)	0.014	0.47	0.39	0.38	0.45
Fat (g)	0.002	0.155	0.8	0.7	0.7
Fiber (g)	0.013	0.2	0.3	0.7	0.4
Water (g)	0.94	0.095	0.12	0.08	0.12
