# Supplementary material for: Diet of schistosome vectors influences infection outcomes
Source: Ecosphere. Author manuscript; Available in PMC 2025 Sep 17. (PMC12439756; doi:10.1002/ecs2.70052)
Supplement: Appendix S4 [file NIHMS2070845-supplement-Appendix_S4.pdf]

Joshua Trapp, Wesley Yu, Johannie M. Spaan, Tom Pennance, Fredrick Rawago, George Ogara, Maurice R. Odiere, Michelle Steinauer. Diet of schistosome vectors influences infection outcomes. Ecosphere.

Appendix S3

Additional analysis (methods and results) of the diet effect on parasite production, while accounting for snail size at time of shedding.

Statistical analysis

Parasite production. A generalized linear model (GLM) with a negative binomial distribution and log link function due to overdispersed, count data was used to determine the effect of diet on parasite production (number of cercariae produced) at 8 weeks post exposure for the compatible KEMRIwu – UNMKenya host-parasite combination, while accounting for the snail size at time of shedding. Similarly for the diet switch experiment, a GLM with a negative binomial distribution and log link function was used to determine the effect of diet switch on the production of parasites at each assessment interval (11-, 14- and 17-weeks post exposure) separately, while accounting for the snail size covariate. The continuous variable, snail size at time of shedding was scaled by standardizing the coefficient estimates (i.e., centering by subtracting the mean of each variable and dividing it by its standard deviation) to ensure that all variables contribute evenly to the scale.

Results

Diet switch effect on parasite production of infection while accounting for snail size at time of shedding

Snail size at time of shedding was relevant to cercarial production at 8 weeks (GLM, β_0 = 4.60, β = 0.45, z = 2.0, p = 0.0463, Appendix S2: Table S1), 14 weeks (GLM, β_0 = 5.37, β = 0.65, z = 2.6, p = 0.0088, Appendix S2: Table S2), and 17 weeks (GLM, β_0 = 4.48, β = 0.66, z = 2.9, p = 0.0043, Appendix S2: Table S2), but not 11 weeks (GLM, β_0 = 4.62, β = 0.29, z = 1.6, p = 0.1078, Appendix S2: Table S2).

Snails switched from a low nutrient to high nutrient diet (lettuce to pellets). At 11 weeks, snails with the switched diet produced 146% more cercariae than high nutrient fed controls (GLM, β_0 = 4.62, β = 0.90, z = 2.5, p = 0.0129, Appendix S2: Table S2). At 14 weeks post-exposure, the number of cercariae produced by the snails with the switched diet (low to high nutrient) peaked and was higher than any other diet group. They produced 293% more cercariae than high nutrient fed controls (GLM, β_0 = 5.37, β = 1.37, z = 2.9, p = 0.0035, Appendix S2: Table S2). By 17 weeks, the number of cercariae produced from snails with the switched diet (low to high nutrient) was similar to both controls; however, sample sizes were small due to mortality (switched diet v. low nutrient diet: GLM, β_0 = 5.42, β = -0.28, z = -0.5, p = 0.5856) (switched diet v. high nutrient diet: GLM, β_0 = 4.48, β = 0.67, z = 1.9, p = 0.0637) (Appendix S2: Table S2).

Snails switched from a high nutrient to low nutrient diet (pellets to lettuce). Surprisingly, the number of cercariae produced by snails switched from a high to low nutrient diet spiked after the diet switch, but then sharply declined over time. At 11 weeks post-exposure these snails produced 97% more cercariae than the high nutrient fed controls (GLM, β_0 = 4.62, β = 0.68, z = 2.5, p = 0.0124, Appendix S2: Table S2). At 14 weeks post exposure, snails switched from a high to low nutrient diet produced the fewest number of cercariae: 91% fewer cercariae than low nutrient fed controls (GLM, β_0 = 6.03, β = -2.42, z = -3.9, p < 0.0001) and 87% fewer cercariae

than high nutrient fed controls (GLM, $\beta_0 = 5.37$, $\beta = -1.76$, $z = -4.1$, $p < 0.0001$) (Appendix S2: Table S2). At 17 weeks, survivorship was poor (only 5 remaining snails in the high to low nutrient diet) and each of the survivors produced 92% fewer cercariae than the low nutrient fed controls (GLM, $\beta_0 = 5.42$ $\beta = -2.50$, $z = -4.3$, $p < 0.0001$), and 79% fewer than high nutrient controls (GLM, $\beta_0 = 4.48$, $\beta = -1.55$, $z = -3.6$, $p = 0.0003$) (Appendix S2: Table S2).

Comparison of control groups: low nutrient v. high nutrient (lettuce v. pellet). The number of cercariae produced by the control groups were not significantly different from each other at each assessment period post diet switch, indicating that the advantage gained by the pellet diet was lost after 8 weeks (GLM, 11 weeks: $\beta_0 = 4.62$, $\beta = 0.34$, $z = 0.7$, $p = 0.4567$; 14 weeks: $\beta_0 = 5.37$, $\beta = 0.65$, $z = 1.0$, $p = 0.3085$; 17 weeks: $\beta_0 = 4.48$, $\beta = 0.94$, $z = 1.6$, $p = 0.1196$; Appendix S2: Table S2).

Table S1 Summary of the generalized linear model output with a negative binomial family, log link function to determine the effect of *Biomphalaria sudanica* KEMRIwu diet (snails fed lettuce vs. pellet) on parasite production (number of cercariae produced) when exposed to *Schistosoma mansoni* (compatible UNMKenya line), while accounting for snail size at time of shedding. Odds ratios represents the back transformed estimates (β). Reference level for diet is lettuce.

	Estimate ($\boldsymbol{\beta}$) ± SE	Odds ratio (CI's)	*z*-value	*p*-value
Intercept ($\boldsymbol{\beta}_0$)	4.60 ± 0.26	99.65 (61.36 – 171.40)	17.7	<0.0001
Diet: Pellet	0.65 ± 0.44	1.91 (0.79 – 4.62)	1.5	0.1465
Snail size at time of shedding*	0.45 ± 0.22	1.56 (1.03 – 2.33)	2.0	**0.0463**

*The continuous variable size was scaled by standardizing the coefficient estimates (i.e., centering by subtracting the mean of each variable and dividing it by its standard deviation) to ensure that all variables contribute evenly to the scale.

Table S2: Summary of the generalized linear model outputs with a negative binomial family, log link function to determine the effect of *Schistosoma mansoni* (compatible UNMKenya line) infected *Biomphalaria sudanica* snails whose diet (lettuce or pellet) got either switched (lettuce to pellet or pellet to lettuce) or remained on the same diet (lettuce control or pellet control) on parasite production (number of cercariae produced) at three different assessment intervals (11-, 14-, and 17-weeks post exposure), while accounting for snail size at time of shedding. Low dietary nutrition represents snails fed green leaf lettuce, whereas high dietary nutrition represents snails fed commercially available pellets (or Aquatic Blended Foods Aquatic Fresh Water Snail Mix). Abbreviations: β_0 = Intercept, Est.= Estimate, SE = standard error, OR = odds ratio, CI's = 95 % confidence intervals.

	**11 weeks post exposure**				**14 weeks post exposure**				**17 weeks post exposure**			
**Comparisons (dietary nutrition):**	β_0	**Est. (β) ± SE**	**OR (CI's)**	***p*-value (*z*-value)**	β_0	**Est. (β) ± SE**	**OR (CI's)**	***p*-value (*z*-value)**	β_0	**Est. (β) ± SE**	**OR (CI's)**	***p*-value (*z*-value)**
low control vs. high control	4.62	0.34 ± 0.46	1.41 (0.59 – 3.44)	0.4567 (0.7)	5.37	0.65 ± 0.64	1.92 (0.54 – 7.08)	0.3085 (1.0)	4.48	0.94 ± 0.61	2.57 (0.77 – 8.88)	0.1196 (1.6)
low to high vs. low control	4.97	0.56 ± 0.36	1.75 (0.86 – 3.53)	0.1250 (1.5)	6.03	0.72 ± 0.56	2.04 (0.67 – 6.17)	0.2044 (1.3)	5.42	-0.28 ± 0.51	0.78 (0.26 – 2.06)	0.5856 (-0.5)
low to high vs. high control	4.62	0.90 ± 0.36	2.46 (1.21 – 5.04)	**0.0129** (2.5)	5.37	1.37 ± 0.47	3.93 (1.55 – 10.26)	**0.0035** (2.9)	4.48	0.67 ± 0.36	1.95 (096 – 3.97)	0.0637 (1.9)
high to low vs. high control	4.62	0.68 ± 0.27	1.97 (1.16 – 3.38)	**0.0124** (2.5)	5.37	-1.76 ± 0.43	0.17 (0.07 – 0.41)	**<0.0001** (-4.1)	4.48	-1.55 ± 0.43	0.21 (0.09 – 0.52)	**0.0003** (-3.6)
high to low vs. low control	4.97	0.33 ± 0.47	1.40 (0.55 – 3.47)	0.4760 (0.7)	6.03	-2.42 ± 0.62	0.09 (0.03 – 0.29)	**<0.0001** (-3.9)	5.42	-2.50 ± 0.57	0.08 (0.03 – 0.25)	**<0.0001** (-4.3)
low to high vs. high to low	5.30	0.22 ± 0.37	1.25 (0.59 – 2.67)	0.5462 (0.6)	3.61	3.13 ± 0.48	22.91 (8.89 – 60.08)	**<0.0001** (6.5)	2.92	2.22 ± 0.43	9.21 (3.82 – 20.98)	**<0.0001** (5.2)
Snail size at time of shedding*	4.62	0.29 ± 0.18	1.33 (0.94 – 1.89)	0.1078 (1.6)	5.37	0.65 ± 0.25	1.91 (1.09 – 3.32)	**0.0088** (2.6)	4.48	0.66 ± 0.23	1.94 (1.16 – 3.24)	**0.0043** (2.9)

*The continuous variable size was scaled by standardizing the coefficient estimates (i.e., centering by subtracting the mean of each variable and dividing it by its standard deviation) to ensure that all variables contribute evenly to the scale.
