# Supplementary material for: Diet of schistosome vectors influences infection outcomes
Source: Ecosphere. Author manuscript; Available in PMC 2025 Sep 17. (PMC12439756; doi:10.1002/ecs2.70052)
Supplement: Appendix S5 [file NIHMS2070845-supplement-Appendix_S5.pdf]

Joshua Trapp, Wesley Yu, Johannie M. Spaan, Tom Pennance, Fredrick Rawago, George Ogara, Maurice R. Odiere, Michelle Steinauer. Diet of schistosome vectors influences infection outcomes. Ecosphere.

Appendix S2

Purpose: Tank effect was explored in the GLMs to support our decision to exclude "Tank ID" as a random effect.

Statistical analysis: For prevalence, snail size at time of shedding, and parasite production, a Kruskal-Wallis rank sum test were used to determine whether there were a tank effect for each diet group separately. The diet switch experiment was the only section that we had repeated measures of the same infected snail (11-, 14-, and 17-weeks post exposure time points). However, we could not distinguish between the individual snails from each diet switch treatment and there was only a single tank per diet switch group. Therefore, for the diet switch experiment we analyzed data from each time point, separately.

Findings:

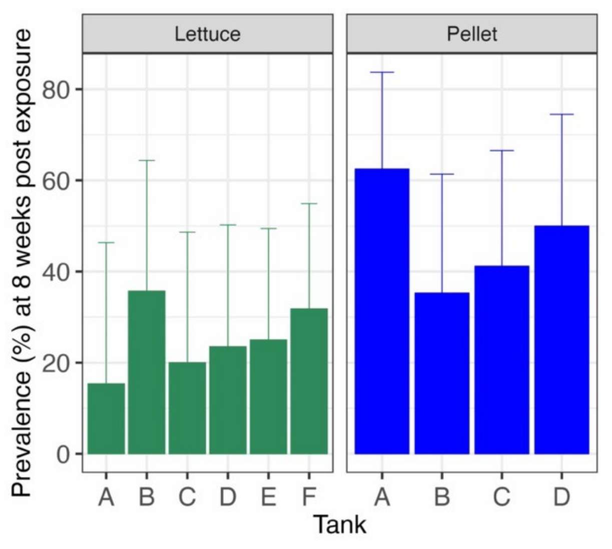

Figure S1 Summary of the prevalence at 8 weeks post exposure for snails fed lettuce (low nutrient diet) in tanks A-F (sea green) and snails fed pellets (high nutrient diet) in tanks A-D (blue). There was no significant difference in prevalence across replicate tanks for lettuce fed snails (Kruskal-Wallis rank sum test, $\chi^2_{df=5}$ = 2.2, P = 0.8253) or pellet fed snails (Kruskal-Wallis rank sum test, $\chi^2_{df=3}$ = 2.7, P = 0.4359). Error bars represent standard deviation of the mean.

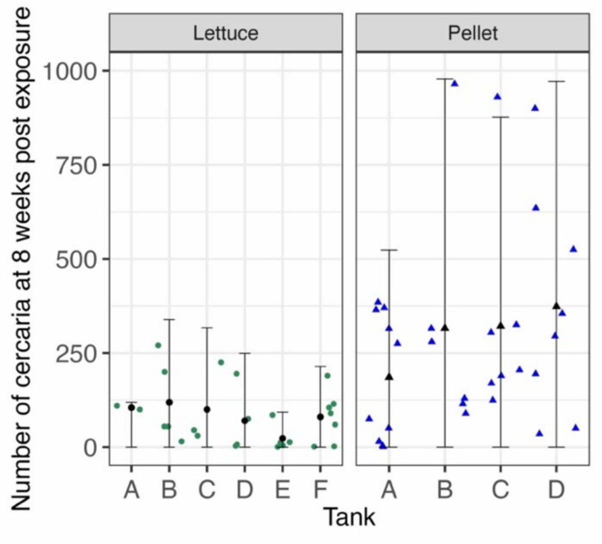

Figure S2: Summary of the number of cercariae shed at 8 weeks post exposure for snails fed lettuce (low nutrient diet) in tanks A-F (sea green, circles) and snails fed pellets (high nutrient diet) in tanks A-D (blue, triangles). There was no significant difference in the number of cercariae shed across tanks for lettuce fed snails (Kruskal-Wallis rank sum test, $\chi^2_{df=5}$ = 5.4, *P* = 0.3697) or pellet fed snails (Kruskal-Wallis rank sum test, $\chi^2_{df=3}$ = 1.9, *P* = 0.6008). Error bars represent standard deviation of the mean.

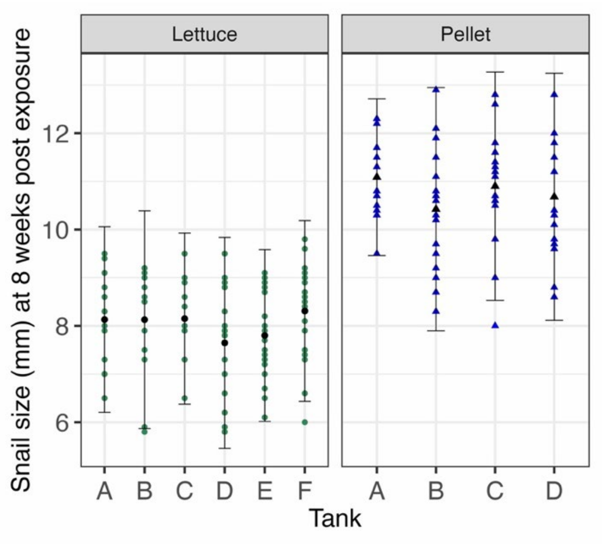

Figure S3: Summary of snail size at 8 weeks post exposure for snails fed lettuce (low nutrient diet) in tanks A-F (sea green, circles) and snails fed pellets (high nutrient diet) in tanks A-D (blue, triangles). There was no significant difference in snail size across tanks for lettuce fed snails (Kruskal-Wallis rank sum test, $\chi^2_{df=5}$ = 6.2, *P* = 0.2858) or pellet fed snails (Kruskal-Wallis rank sum test, $\chi^2_{df=3}$ = 3.3, *P* = 0.3440). Error bars represent standard deviation of the mean.

Conclusion: There was no replicate tank effect for prevalence, snail size at time of shedding, or parasite production, for either diet group. These findings supported our decision to exclude "Tank ID" as a random effect.
